# Physical Activity and Brain Plasticity

**DOI:** 10.20463/jenb.2019.0027

**Published:** 2019-12-31

**Authors:** Hyo Youl Moon, Henriette van Praag

**Affiliations:** 1 Dept. of Physical Education, Seoul National University, Seoul Republic of Korea; 2 Institute of Sport Science, Seoul National University, Seoul Republic of Korea; 3 School of Biological Sciences, Seoul National University, Seoul Korea; 4 Department of Biomedical Science, Florida Atlantic University, Jupiter USA

**Keywords:** Physical activity, Neurogenesis, Brain plasticity, Hippocampus

## Abstract

Recent research suggests that the brain has capable of remarkable plasticity and physical activity can enhance it. In this editorial letter, we summarize the role of hippocampal plasticity in brain functions. Furthermore, we briefly sketched the factors and mechanisms of motion that influence brain plasticity. We conclude that physical activity can be an encouraging intervention for brain restoration through neuronal plasticity. At the same time, we suggest that a mechanistic understanding of the beneficial effects of exercise should be accompanied in future studies.

Studies have demonstrated that physical activity affects brain plasticity, cognition and mood^1-4^. Indeed, animal experiments and clinical studies show tremendous biological and psychological benefits of physical activity, and accompanying structural and functional changes in the various brain regions^5,6^. In recent years, the effect of physical activity on memory improvement in neurodegenerative diseases patients has attracted attention^7^. The circuits of the limbic system are known to regulate learning and memory function^8,9^. Subjects with cognitive impairment have been shown to have reduced volume in the hippocampus and forebrain^10-12^. Adult neurons have been thought to be unable to be replaced by new cells because cell division is over, but recent studies have found that new neurons are born (neurogenesis) in select regions of the adult brain and may contribute to maintainance of neuronal function^13^. Experiments utilizing the thymidine analog bromodeoxyuridine (BrdU) to label dividing cells and several genetic markers have revealed that hippocampal dentate gyrus is one of the brain regions showing the neurogenesis in mature animals, and this was conserved in rodents, primates, and humans^13,14^. Adult neurogenesis was also proven in bird studies; a comparison of hippocampus size and number of neurons with a long-traveled migratory bird and a non-traveling migratory bird also shows that hippocampus may be important for memory and experience^15^. In addition, chickadees exhibit enhanced neurogenesis when they store seeds for winter^16^.

Neuronal plasticity is key feature for the cognition and it is regulated by neurogenesis, synapse formation, angiogenesis and changes in neurotransmitter system^3,6,9^. Animal experiments using voluntary wheel cages and treadmills have reported that exercise increases the proliferation of neurons in the hippocampus of rodents^3^. Exercise induces the changes of the neurotransmitter systems such as serotonin and acetylcholine and the release of factors such as BDNF and IGF-^12,17^. Along with these changes, exercise improves the cognitive functions such as spatial and executive functions^18,19^. These changes may also be very effective interventions in aging and degenerative brain disease models^20,21^.

Recent studies have shown that exercise promotes the release of factors such as peripheral BDNF^22^, IRSIN^23^, IGF^24^, and Cathepsin B^5^, which are systemically delivered to the brain and may play a role in cognitive function. Furthermore, we found that conditioned media which containing secreted proteins from skeletal muscle cells could influence adult hippocampal neural progenitor cell (aNPC) differentiation^25^. Exercise induced neurogenesis can be also affected through epigenetic modifications^26^ or the balance of intestinal microflora^27^. In fact, even though the mechanisms are not clearly investigated, there are reports demonstrating exercise affects intestinal microorganisms^28^ or brain epigenetics^29^. There has been little research on the effects of metabolites on the brain and nerve system. Studies have shown that these metabolites are not only end products during metabolism but also act as hormones in a variety of physiological and pathological conditions as a signaling material^30,31^. In the near future, exercise-induced changes in these biological markers may be the candidate target of new exercise mimetics and may play an important role in proposing or prescribing exercise appropriate to the individual's health status. Most of the cross sectional studies investigating changes in the human brain after exercise have limitations in observing the neuronal adaptation during or after exercise. Future studies are needed to understand the effects and mechanisms of exercise from a systemic and longitudinal view. Moreover, human studies are needed to show that exercise is important for the learning and memory function via hippocampal neurogenesis.
